# Requirements to justify breastfeeding in public: a philosophical analysis

**DOI:** 10.1186/s13006-019-0217-x

**Published:** 2019-06-12

**Authors:** Fiona Woollard

**Affiliations:** Philosophy, University of Southampton, Southampton, England UK

**Keywords:** Breastfeeding in public, Right to breastfeed, Philosophy, Ethics of infant feeding

## Abstract

**Background:**

It may be tempting for breastfeeding advocates to respond to challenges of breastfeeding older children or breastfeeding in public, by pointing out the nutritional or developmental benefits of breastfeeding, or by noting that breastfeeding is often extremely discreet. Such responses may concede more than they should: by focusing on rebutting the empirical claim, breastfeeding supporters may end up implicitly accepting two presuppositions about breastfeeding. First, the presupposition that breastfeeding requires justification in terms of health or developmental benefits to the child, and second, the presupposition that breastfeeding in public is only acceptable if assumed standards of discretion are met.

**Discussion:**

This paper aims to use the methods of analytic philosophy to: (a) show how, if left unchallenged, these implicit assumptions can become part of the pragmatic presuppositions of the conversation, so that the discussion proceeds taking their acceptance for granted, (b) argue that we can expect these presuppositions to have negative effects on all mothers, no matter how they feed their babies, and on the tenor of public discussion of infant feeding, (c) reconstruct the reasoning that might underlie these presuppositions and show that this reasoning is mistaken, and (d) show that recognising breastfeeding as a family way of life and a loving interaction between parent and child gives rise to a moral right to breastfeed in public without social sanction, whether one is able to breastfeed discreetly or not.

**Conclusions:**

Mothers have an unconditional, moral right to breastfeed and to feel welcome to breastfeed in public even if they are not able to breastfeed ‘discreetly’.

## Background

In January 2015, ITV’s *This Morning* ran a segment on natural term breastfeeding, featuring a mother who was “still” breastfeeding her six-year-old daughter. Clare Byam-Cook also appeared on the programme as a “breastfeeding specialist” and is quoted as saying:

*‘The nutritional benefits at six are virtually negligible, and also you say that you breastfeed your six-year-old when she’s tired or needs comforting, so you’re teaching your child to use food as a source of comfort.*

*Why can’t you just cuddle her? The father doesn’t have to breastfeed to comfort, so it just seems to me it’s the wrong association. I don’t think it’s natural in this country’* [1].

Roughly two years later, in March 2017, *This Morning* again aired a debate on breastfeeding, this time asking viewers: “Is it OK to breastfeed in a pub?” Although most of the discussion was supportive of public breastfeeding, one guest, freelance journalist and fitness expert, Nilufer Atik, argued:

‘*I’m not against breastfeeding in public, I just think women can use their discretion because not everybody is comfortable with it and we shouldn’t expect them to*. *.*. *I’ve had this conversation with some of my male friends and they say if a woman walks into a pub and she’s got her cleavage on display, it’s a man’s nature to look. They can’t help it, it’s biological for a heterosexual man to look’* [2].

Supporters of breastfeeding might be tempted to respond to Byam-Cook’s attack on natural term breastfeeding by pointing out that breastmilk does not suddenly lose its nutritional benefits when a child passes an arbitrary age [3–5]. They may wish to respond to Atik’s concerns about breastfeeding in public by pointing out that breastfeeding is normally discreet. Both presenters of *This Morning* responded to Atik in this way.

## Discussion

In this paper, I show that such responses concede more than they should: by focusing on rebutting the empirical claim, breastfeeding supporters may end up implicitly accepting two presuppositions. First, the presupposition that breastfeeding requires justification in terms of health or developmental benefits to the child and second, the presupposition that breastfeeding in public is only acceptable if assumed standards of discretion are met.

This paper uses the methods of analytic philosophy to explore how these two presuppositions may affect discussion of the acceptability of breastfeeding. I aim to, (a) show how, if left unchallenged, these implicit assumptions can become part of the pragmatic presuppositions of the conversation, so that the discussion proceeds taking their acceptance for granted, (b) argue that we can expect these presuppositions to have negative effects on all mothers, no matter how they feed their babies, and on the tenor of public discussion of infant feeding, (c) reconstruct the reasoning that might underlie these presuppositions and show that this reasoning is mistaken and, (d) show that recognising breastfeeding as a family way of life and a loving interaction between parent and child gives rise to a moral right to breastfeed in public without social sanction, whether one is able to breastfeed discreetly or not.

### Presuppositions about when breastfeeding is acceptable

The philosophical understanding of a pragmatic presupposition was first proposed by Robert Stalnaker and later developed by David Lewis amongst others [6, 7]. Pragmatic presuppositions are the propositions that are taken for granted during a conversation, even though they may not follow from the literal meaning of what is said. It is assumed, or purported to be assumed, that these presuppositions are common knowledge, that they ‘go without saying’. Pragmatic presuppositions develop during a conversation. If one of the speakers says something with a given presupposition, and this is not challenged by the other speakers, this presupposition becomes part of the conversation’s pragmatic presuppositions. For example, suppose that I say “When Mary goes to the store to buy more tea, she should take the car.” You might reply that Mary should walk instead. Although you are challenging my recommendation that Mary should take the car, you have left unchallenged, and thus implicitly accepted, the presuppositions that more tea is desirable, that going to the store is an appropriate way to get more tea and that Mary will be the person to do this. These claims now become part of the pragmatic presuppositions of our conversation and the conversation goes forwards on the assumption that all parties agree about the desirability of Mary buying more tea.

Suppose someone claims that breastfeeding at a given age is inappropriate because there are no nutritional or developmental benefits at that age. If our response focuses on pointing out nutritional or developmental benefits, then we are implicitly accepting the presupposition that breastfeeding is inappropriate unless it has such benefits. We are implicitly accepting the presupposition that breastfeeding is something that requires justification. Similarly, if we respond to complaints about breastfeeding in public solely by pointing out that very often it is not even possible to tell that a mother is breastfeeding, we are implicitly accepting that breastfeeding in public is only acceptable when certain standards of discretion are met. These claims now become part of the pragmatic presuppositions of our conversation, the conversation goes forward on the assumption that all parties agree with them.

Empirical studies give some evidence of widespread acceptance of these two presuppositions in a variety of geographical locations. See for example, Elizabeth Murphy’s work showing that pregnant women who intended to breastfeed felt required to respond to charges that their intention to breastfeed involved deviant behaviour: “breastfeeding was treated as potentially problematic for mothers’ status as moral and decent women” [8]. Empirical studies of public attitudes towards breastfeeding provide further evidence for my suggestion that often breastfeeding is seen as only acceptable if it is discreet [9–11].

Some of these studies date back as far as the early 1990s. We might hope that in 2019 that there would be much wider acceptance of public breastfeeding. However, the acceptability of public breastfeeding was still being debated on *This Morning,* one of the UK’s flagship morning television programmes, in 2017. In this debate the necessity of discretion was generally unchallenged as a presupposition of the conversation and defences of public breastfeeding typically focused on how little flesh would standardly be revealed during breastfeeding. This suggests that the attitude of at most conditional acceptance of breastfeeding is still common.

### Expected effects of the presuppositions

I have identified two presuppositions which may be implicitly accepted in discussion of breastfeeding past infancy and breastfeeding in public. First, the presupposition that breastfeeding requires justification in terms of health or developmental benefits to the child and second, the presupposition that breastfeeding in public is only acceptable if assumed standards of discretion are met. Both these presuppositions can be expected to have negative effects on all mothers, no matter how they feed their babies, and on the tenor of public discussion of infant feeding.

Breastfeeding is a significant activity for new mothers. Mothers, particularly mothers of newborns, may spend hours each day breastfeeding. They may have overcome considerable difficulties in order to breastfeed. Breastfeeding may be seen as an important aspect of their relationship with their child, involving both physical and emotional intimacy. The assumptions treat this extremely significant activity as potentially deviant. They make the mother’s daily activity, involving the most intimate aspects of her relationship of her child, subject to the scrutiny of others. If the mother avoids breastfeeding in public, her ability to live a normal life will be severely curtailed. If the mother breastfeeds in public, she must worry about whether she is living up to the standards of discretion. Failure to do so is taken to cast doubt on her modesty and her respect for others.

Being subject to this kind of moral surveillance is bad for the mother’s wellbeing in its own right. Moreover, it can be expected to contribute to negative emotions about breastfeeding, particularly shame or embarrassment about breastfeeding in public. Again, these negative emotions are in themselves harmful and should be a concern insofar as we care for women’s wellbeing, but they can also have further bad effects. Lisa Amir, Jacqueline H. Wolf, and Kate Boyer raise concerns that worry about embarrassment can prevent women from breastfeeding or contribute to women feeling unable to continue breastfeeding [12–14]. Several studies identify discomfort with breastfeeding in public as a contributing factor in shaping infant feeding decisions and the decision to stop breastfeeding in particular [15, 16]. For discussion of this evidence see Boyer [14].

The requirement to meet standards of discretion may also lead mothers to breastfeed in ways that cause physical harm. As Amir comments “Covering the breasts during feeding has implications for maternal and infant health and well-being. In my clinical practice, I’ve seen a woman who developed mastitis after feeding awkwardly because she was concealing her breast in a public setting” [12].

Treating breastfeeding as requiring justification may also be expected to contribute to perceived division between mothers who breastfeed and mothers who use formula. Mothers may end up feeling that in order to show that their own conduct is acceptable, they need to show that other feeding methods are unacceptable. A tempting way to respond to the charge that one’s breastfeeding behaviour is deviant is to argue that avoiding breastfeeding is not a real option. If a mother can show that she *has* to breastfeed, she can avoid accusations that by breastfeeding she is failing to be sufficiently considerate, modest or discreet. She may do this by arguing that the health and developmental benefits produce a defeasible duty to breastfeed (mothers are required to breastfeed unless there are sufficiently weighty countervailing considerations). Unfortunately, this strategy has negative implications for mothers who do not breastfeed. Failure to live up to defeasible duties entails liability to be called on to justify one’s behaviour and to guilt and blame if one doesn’t have a good enough excuse. So, holding that there is a defeasible or absolute duty to breastfeed leaves mothers who do not breastfeed facing guilt and blame. I’ve argued elsewhere that these are both unwarranted and harmful for mothers who do not breastfeed and their neonates [17].

Thus, the idea that breastfeeding is a potentially deviant activity which requires justification can be expected to contribute to a false picture of ‘breastfeeding mothers’ and ‘formula feeding mothers’ as separate and antagonistic groups. This is bad for all mothers, however they feed their babies. Antagonism between mothers disrupts potential networks of support and collaboration. Moreover, mothers may not fall neatly into just one of these groups. Some mothers will both breastfeed and use infant formula, either mixed feeding one baby or making different feeding decisions with subsequent children. When women fall into both groups, the idea that defending one group requires us to condemn the other is particularly unhelpful. Finally, pitting breastfeeding mothers against mothers who use formula can be expected to undermine our ability to have fruitful conversations about infant feeding practice and policy. It can be expected to contribute to a climate where actions designed to support breastfeeding are felt as an attack on mothers who use formula and vice versa.

### Reconstruction and refutation of the reasoning behind the presuppositions

Most of our behaviour does not have to provide health or developmental benefits in order to make it acceptable. It sounds very odd to say “You should not feed your child porridge after they are five, they can get all the same nutrients from a balanced diet that doesn’t include porridge” or “You should not stroke your child’s hair, they can get all they need from other methods of showing affection” or “You should not tell stories to your child when they are old enough to read to themselves. At that point, it has no developmental benefit.” Of course, like many of things that we instinctively do with our children, telling stories may well provide developmental benefits that children cannot acquire by reading alone. The key point is that the acceptability of the practice does not depend upon the existence of such benefits. It is enough that this is something that the parent and child want to do together. The default position is for our behaviour to be acceptable without need for further justification. This is for good reason, as being required to justify all of our everyday practices would be a stifling form of moral scrutiny.

That is not to say that our behaviour is never appropriately subject to moral scrutiny. As I argue elsewhere, requiring people to justify their behaviour, to provide assurance that they haven’t behaved badly when there is reason to think that they may have done so, is a crucial part of our moral practice [18]. However, this reassurance is only required when there is reason to think that the behaviour may be wrongful. Justification is only required for behaviour which is potentially deviant.

Breastfeeding may be seen as potentially deviant, and thus requiring justification, due to (a) concerns about causing others to feel discomfort, embarrassment or sexual arousal, (b) ideals of feminine modesty, and (c) worries about inappropriate contact between mothers and children. One strong theme underlying these concerns is the view of the female breast as predominantly a sexual body part. One way to respond to the idea that breasts are primarily sexual is by arguing that their primary evolutionary role is to nurture babies. However, this response does not make room for women who do wish to see their breasts as sexual [19]. A better response may be to argue that whether a woman’s breast is sexual at a given time should depend on what the woman is *doing* with it at that time. Breastfeeding one’s child is not a sexual activity. When a woman is using her breasts to breastfeed, they are not predominantly sexual body parts.

Another underlying theme is the idea of breastfeeding as analogous to other activities which normally take place in private, either as a bodily function analogous to other bodily functions like urination [12, 20] or as an intimate activity analogous to other intimate activities like sex. Such analogies ignore the unique nature of breastfeeding. Breastfeeding is a natural bodily function involving the secretion of bodily fluids, however, those fluids are not waste but food. Breastfeeding can be an extremely intimate act, but it is also a mundane part of childcare, which may need to take place frequently throughout the day. It is also a wonderful way of providing the comfort and reassurance that a child may need to cope with the challenges that they meet in their daily adventures.

Given all this, none of the identified reasons for treating breastfeeding as in need of justification stand up to scrutiny. It is questionable whether failing to live up to feminine ideals of modesty should make a behaviour potentially deviant and in need of justification. There’s good reason to think that we should reject these ideals of modesty altogether. But even if we do accept the ideal of feminine modesty, breastfeeding when properly conceived is not in conflict with these ideals. Because breastfeeding is not fully analogous to bodily functions like urination or to intimate activities like sex, it is not an activity which modesty requires us to do in private. This is not to discount the feelings of those women who do prefer privacy for breastfeeding, and a preference for privacy is perfectly reasonable. Instead, it is to say that women who are comfortable breastfeeding in public are not violating a coherent and justifiable norm of modesty.

Similarly, worries about inappropriate contact between mother and child should be dismissed when we recognise that breastfeeding a child is not a sexual act. The idea that pleasurable contact with a woman’s breasts is by default sexual is powerful. This idea makes us see breastfeeding as involving contact that is by default inappropriate and can only be justified if it is necessary for the child’s health or development. It is partly linked to an idea that pleasurable contact with another’s body is in general sexual. The philosopher, Alan Goldman, argued that a desire counts as sexual if and only if it is a desire for pleasurable contact with another person’s body for its own sake [21]. But normal love for one’s child is both non-sexual and deeply embodied and holding our children is not just a neutral means for conveying affection; the softness, weight and even the smell of our babies is a pleasure in itself. Once we recognise that neither the breast itself nor pleasurable physical contact with another’s body need be sexual, we can see that breastfeeding is not, by default, inappropriate contact between mother and child.

We now move to concerns about causing others to feel discomfort, embarrassment or sexual arousal. It might seem that the fact that an action may make others uncomfortable does make the action potentially deviant and requiring justification. Surely, we shouldn’t make other people feel bad unless we have to?

But the mere fact that my behaviour will make other people feel bad cannot by itself make my behaviour potentially deviant. That kind of view makes me vulnerable to arbitrary restrictions on my freedom through the whims of others. Homophobic people may feel uncomfortable if they see a gay couple holding hands. That doesn’t mean that the gay couple should only hold hands if they have to. Discomfort about seeing breastfeeding in public or being near gay people holding hands seems to be connected to what Ronald Dworkin called an ‘external preference’. A personal preference is a preference about what happens to me. An external preference is a preference about what happens to other people [22]. My preferences about what happens to me are morally important in a way that my preferences about what happens to other people are not.

The spectator might argue that something is happening to him. After all, *he* is being forced to see something. Nonetheless, it is an external preference in a relevant sense. The breastfeeding mother and the gay couple are not really doing anything *to* the spectator. Their behaviour does not primarily concern him. It only causes him discomfort because of his attitudes towards their behaviour. I call such preferences ‘primarily external preferences’.

We do sometimes give weight to people’s preferences about what they should be confronted with in their daily lives. Many states have laws forbidding ‘flashing’, public nudity, and public urination. Flashing is the intentional display of sexual organs to someone without their consent. It is different from public nudity because part of what is aimed at is the reaction of the victim. Because an effect on the victim is a key aim of the behaviour, flashing can be thought of as doing something to the victim. Flashing over-rides the victim’s *personal* preferences. Indeed, it violates her personal *sexual* preferences. Because respect of personal sexual preferences is a particularly important aspect of autonomy, this is rightly treated as a serious wrong.

In contrast, preferences about public nudity and urination (where the urine is contained) are primarily external preferences. When we do give weight to such preferences it tends to be because we as a society endorse the view that the thing that the person prefers not to be confronted with is something that preferably should not occur in public *and* the cost of treating behaviour that goes against the preference as potentially deviant and requiring justification is not too high. Neither of these conditions applies in the case of discomfort about breastfeeding. I will argue that the cost of treating breastfeeding in public as potentially deviant is extremely high and the mother has a moral right to breastfeed in public which rules out treating breastfeeding in public as requiring justification. In addition, the considerations raised show that we should not endorse the view of breastfeeding as something that preferably should not take place in public. As I argued above, breastfeeding is neither sexual nor analogous to ‘private’ bodily functions like excretion. There is no reason to see breastfeeding in public as inappropriate.

### The moral right to breastfeed – and all it entails

I have discussed three possible concerns that might be behind the presupposition that breastfeeding is potentially deviant and requires justification or that is only permissible if it is discreet. I have argued that these concerns do not give us reason to treat breastfeeding as requiring justification. I now argue further that considerations about the importance of intimate family relationships warrant a moral right to breastfeed that is independent of any health or developmental benefits of breastfeeding and entails a moral right to breastfeed in public without social sanction, whether one is able to breastfeed discreetly or not.

The moral right to breastfeed does not depend upon any health or developmental benefits of breastfeeding. Instead, it is part of (a) the moral right to pursue our own family ways of life, and (b) the moral right to intimacy between parent and child. I understand a way of life to be a significant aspect of how a person, family or community organises and understands their lives and relationships. The term ‘way of life’ is strongly associated with Alasdair MacIntyre who argued that the good life for any individual cannot be understood in a vacuum, that understanding what is good for me requires an understanding of the culture, traditions, and community that surround me [23]. I use this term with a deliberate nod to MacIntyre, to emphasise that ways of life, as well as reflecting deeply personal values, are very often embedded in cultural or family traditions. An individual’s way of life might include going to church every Sunday or adhering to a vegetarian diet. I use the term ‘a family way of life’ to indicate a significant aspect of how one organises one’s family, the key practices and values that shape the relationships between family members and how the family unit functions as a unit. This includes the organisation of daily life as well as landmark events.

It is deeply important to both parents and children to be able to pursue their own family ways of life. Family relationships, like the relationship between parent and child, are a key part of human life. The ability to make decisions about one’s family’s way of life based on one’s own values and traditions is a fundamental part of our autonomy.

Philosophers such as John Feinberg have emphasised that parents’ moral right to bring their children up to hold a certain worldview is limited. Feinberg argues that children have a right to an open future and children must not be brought up in such a way that they are prevented from exercising choice [24]. However, this debate is almost always focused on what, if any, limits there are to parents’ right to choose how to bring up their children. It is generally assumed that there is a defeasible right to make important decisions about one’s family life and the puzzle is about what we should do when parents appear to wish to exercise this right in ways that are worrying for other reasons. Thus, for example, Feinberg argues that a parent’s right to make decisions for their family does not extend to a religious-based exemption to requirements for the child to attend school. In making this argument, Feinberg appeals to the child’s right to an open future as a limit on parental rights. The need to appeal to such a consideration shows that it is assumed that families are entitled to arrange their family life according to their traditions and values *unless there are some significant opposing considerations*.

So, when I say that there is a moral right to pursue one’s family way of life, I do not mean that all families are automatically entitled to resources to arrange their family life as they wish or that no considerations of effects on others are relevant. It is permissible to have rules restricting noise after 11 pm, even if this is incompatible with a nocturnal family way of life. Instead, I mean that there is a default entitlement to arrange the key practices and values of one’s family and that, as far as is reasonable, society should be set up to enable people to pursue their preferred family ways of life.

Decisions about whether, and how, to breastfeed are decisions about what your child will eat, how you will comfort your child and how you will help your child to sleep. Breastfeeding is a family way of life. It clearly does not undermine the child’s right to an open future and I have argued above that there are no other important reasons for concern. Therefore, the moral right to pursue one’s own family way of life includes a right to breastfeed.

This argument for a moral right to breastfeed is similar to Lisa Smyth’s argument that infant feeding should be regarded as ‘a site of intimate citizenship’ [16]. Smyth’s argument draws on Martha Nussbaum’s capabilities account of human rights, that all citizens should be enabled to develop the full range of their capacities as human beings, including most importantly, our autonomy, the ability to ‘direct our own lives’ [25]. Smyth argues that breastfeeding involves exercising our ability to direct our own lives according to our conception of ‘the good’. For this reason, “Public policy on the intimate citizenship practice of breastfeeding should enable women and men to make well-informed, and highly personal decisions about how to care for their infants in ways that enhance rather than diminish their sense of autonomous self-hood” [16]. The Smyth/Nussbaum approach provides an excellent way of thinking about what is at stake when we think about the moral right to breastfeed. Nonetheless, my argument does not depend on acceptance of Nussbaum’s account of human rights. A moral right to breastfeed should be recognised by any account that recognises, (a) autonomy as a basic human good that demands a default entitlement to make decisions about key areas of our lives, and (b) decisions about breastfeeding as decisions that have significant impact on family life and that connect deeply to parents’ values and culture. Surely any satisfactory account of our basic moral rights should recognise both these things.

Decisions about whether to breastfeed are also decisions about whether to share a physical and physiological union that can express a deeply embodied love. Parents and children have a moral right to engage in such loving interactions. Indeed, I suggest that, unless there is reason for concern about inappropriateness, parents and children have the moral right not just to engage in some loving interactions, but to engage in their preferred form of loving interactions. They have the moral right to hug, to hold hands, to touch noses or whatever their practice is. However, even if this expansive right is rejected, we should support a moral right for mothers and children to breastfeed as a loving interaction. The decision to breastfeed may not feel like a decision at all. It may seem like simply a response to a deep instinct to nurture your child in the way mammals have evolved to nurture their young. A mother and child may never breastfeed and yet have a thousand embodied loving interactions every day. Nonetheless, mothers and children surely have a right to this particular form of loving interaction if it is desired by both mother and child.

Could we recognise the moral right to breastfeed, while restricting the right to breastfeed at a given time or place? No. Restrictions on public breastfeeding, including social sanctions for those who are perceived as failing to feed ‘discreetly’, force mothers to make a choice between breastfeeding, the physical and emotional comfort of themselves and their children, and full participation in public life. As Wolf notes, many critics of public breastfeeding have an insufficient grasp of how breastfeeding works and therefore do not appreciate exactly what a restriction on breastfeeding in public implies for the breastfeeding mother:

*.. . “In the U.S., people who oppose breastfeeding in public often argue, ‘What’s the problem? Feed the baby before you leave the house. There’s no reason, with a little planning, to breastfeed in a restaurant or at the mall.’ This insistence that babies should only be breastfed behind closed doors demonstrates a fundamental lack of understanding of both the composition of human milk and babies’ needs. Babies have to nurse while they are out and about due to the nature of human milk”* [13].

Breastfed babies, especially when very young, need to feed frequently. Each feed may also take a significant amount of time. Requiring mothers of young babies to feed before they leave the house or to hide away in lactation rooms effectively requires them to spend most of their time in isolation. Waiting long periods of time between feeds not only leaves the baby suffering from hunger, it may also leave the mother suffering from painfully engorged breasts or even at risk of mastitis. Nor is it simple to just switch to give a bottle when out in public. A breastfed baby may refuse to bottle-feed. A breastfeeding mother may be reluctant to give her baby infant formula milk. Expressing milk is extremely time consuming and some women do not respond well to the breast pump, even when they are able to produce plentiful milk for their baby when he feeds directly. Older babies and children may be able to wait longer to satisfy their hunger, but if they breastfeed, they may wish to breastfeed in public on occasion for comfort or reassurance. Some breastfeeding women do use expressed milk or formula or allow older children to breastfeed only in private. However, whether these things are possible without undermining breastfeeding will vary from dyad to dyad and within the same dyad at different stages. This should not be a condition of the right to a breastfeeding relationship.

The answer to the need to breastfeed in public cannot be a conditional acceptance, such that breastfeeding in public is acceptable only if it is discreet. As argued above, attempts to live up to assumed standards of discretion cause breastfeeding mothers physical harm and lead to shame, guilt and embarrassment. There may be many reasons that a mother finds it difficult to breastfeed discreetly, from an easily distracted child to the size of her areolas. Mothers have a right to make decisions about their family way of life and to loving interactions with their child whether or not they are able to breastfeed discreetly. The conditional acceptance of breastfeeding *only if* it is discreet, forces mothers who are not able to breastfeed discreetly to suffer physical or emotional discomfort, or allow their children to do so, or forfeit full participation in public life in order to exercise their moral right to breastfeed.

Under these conditions, a mother may end up sacrificing either her moral right to breastfeed or her right to participation in public life. The paper from Smyth, which I discussed above, shows that both of these choices conflict with women’s status as citizens. As well as arguing that opportunities to breastfeed,and more generally to choose how and where to feed one’s babies, are themselves a key part of ‘intimate citizenship’, Smyth explores the way in which restrictions on breastfeeding in public raises issues of gendered assumptions about the use of social space which may limit women’s access to citizenship. She says: “.. . breastfeeding, would seem to offer a good example of where citizenship, in this case intimate, is mediated by a gendered entitlement to inhabit and use public space” [16]. This part of Smyth’s argument draws two key ideas from existing work challenging the gendered nature of citizenship. First, citizenship, defined as a sense of belonging, depends on the ability to use collective spaces, and second, women’s access to this kind of citizenship is severely compromised by their gender in interaction with other social divisions such as race [16]. When it is not seen as acceptable for women to access public spaces while visibly breastfeeding or to visibly breastfeed while accessing public spaces, the message is that the ‘public’ for whom those spaces exist does not include those with messy, female bodies and caregiving responsibilities. See Jennie Munday [26] for an excellent overview of ways in which allegedly gender-neutral traditional conceptions of citizenship exclude women.

It is unreasonable to require *any* mother to suffer physical or emotional discomfort, or allow their children to do so, or forfeit full participation in public life in order to exercise their moral right to breastfeed. Thus, the moral right to breastfeed entails an *unconditional* moral right to breastfeed in public without social sanction. By saying that this right is ‘unconditional’, I mean that it does not depend upon being able to meet any given standards of ‘discretion’.

## Conclusions

I identified two presuppositions which may be left unchallenged in discussion of breastfeeding past infancy and breastfeeding in public. The first presupposition is that breastfeeding requires justification in terms of health or developmental benefits to the child. The second presupposition is that breastfeeding in public is only acceptable if assumed standards of discretion are met. Both these presuppositions can be expected to have negative effects on all mothers, no matter how they feed their babies, and on the tenor of public discussion of infant feeding.

I have reconstructed the reasoning that may lie behind acceptance of these presuppositions and argued that it is mistaken. Breastfeeding is not a potentially deviant activity requiring justification. On the contrary, there is a moral right to breastfeed grounded in the moral right to pursue our own family ways of life and the moral right to intimacy between parent and child. This moral right to breastfeed entails an unconditional right to breastfeed in public without social sanction. Mothers should be made to feel welcome to breastfeed in public even if they are not able to breastfeed ‘discreetly’.

## Data Availability

Not applicable
